# Will Music Give Me Power? Effects of Listening to Music during Active and Passive Rest Intervals on Power Output during Resistance Exercise

**DOI:** 10.3390/jfmk9010032

**Published:** 2024-02-16

**Authors:** Agata Latocha, Jakub Jarosz, Jonatan Helbin, Michał Krzysztofik

**Affiliations:** 1 Nutrition and Sports Performance Research Group, The Jerzy Kukuczka Academy of Physical Education in Katowice, 40-065 Katowice, Poland; agatalatocha1@gmail.com (A.L.); j.jarosz@awf.katowice.pl (J.J.); jonatan.helbin@gmail.com (J.H.); 2 Institute of Sports Sciences, The Jerzy Kukuczka Academy of Physical Education in Katowice, Mikołowska 72a, 40-065 Katowice, Poland

**Keywords:** resistance training, music effect, motivation, focus, bench press, back squat

## Abstract

The study aimed to evaluate the impact of listening to preferred music during active/passive rest on power output and heart rate in barbell squats (BS) and bench presses (BP). Fifteen participants (13 males and 2 females), moderately resistance trained, were engaged in four randomized experimental sessions with varying rest intervals (active/passive) and music presence (listening or not). Each session involved three sets of three repetitions of BS and BP at a 50% one-repetition maximum. ANOVA showed a significant main effect of the set for BP relative mean and peak power output (*p* < 0.001; both). The post hoc comparisons indicated a significantly higher BP relative mean and peak power output in set_2 (*p* < 0.001; effect size [ES] = 0.12 and *p* < 0.001; ES = 0.10) and set_3 (*p* < 0.001; ES = 0.11 and *p* = 0.001; ES = 0.16) in comparison to set_1. Moreover, a main effect of the set indicating a decrease in BS relative peak power output across sets was observed (*p* = 0.024) with no significant differences between sets. A significantly higher mean heart rate during active rest in comparison to passive rest was observed (*p* = 0.032; ES = 0.69). The results revealed no significant effect of listening to music on relative power output and heart rate during BS and BP.

## 1. Introduction

Resistance training has been utilized for years in improving overall and specific physical fitness, injury prevention and rehabilitation protocols [1]. Back squats (BS) and bench presses (BP) are basic exercises that have become a permanent part of exercise programs. Both exercises are part of powerlifting competitions and are commonly used by athletes practicing many sports. The BS is one of the exercises with the greatest potential to increase the development of strength, power, and overall athletic performance of the lower limbs [2], while the BP is the most common exercise to increase upper body strength [3].

To enhance sports performance and stagnation, individuals and coaches employ various training methods (beyond traditional resistance training) and ergogenic aids including supplements as well as verbal motivation or music [4,5]. Music plays a significant role in everyday life, both on an emotional and physical level [6]. The influence of music encompasses psychological [7], psychophysical, and physiological factors [8], which focus on how music affects mood, emotions, feelings (a sense of pleasure or displeasure) [9], cognition (thought processes) [10], behavior [11,12], physical exertion [13], and cardiovascular response [14]. 

The impact of music has been extensively studied, and numerous evidence has confirmed the use of music as an ergogenic aid during physical activity [15]. A growing body of research has demonstrated the impact of music on physiological variables, including blood pressure, heart rate respiration rate, body temperature [16], and biochemical parameters, in addition to pain sensitivity [17]. Furthermore, music has a positive effect on athletic performance, by delaying fatigue and increasing work capacity [18]. In the context of resistance training, research also indicates an ergogenic effect of listening to music. For instance, Cutrufello et al. [19] revealed that compared to conditions without music, listening to self-selected music resulted in a significant enhancement in the total number of BP repetitions at 70% one-repetition maximum (1RM) among healthy, college-aged students. Similarly, Ballmann et al. [20] observed an increase in the total number of BP repetitions at 75%1RM after a warm-up session during which preferred music was listened to, as compared to non-preferred music. However, the authors did not find differences in barbell velocity between conditions. Conversely, the subsequent study by these authors found that listening to music before BP significantly increased barbell velocity, as well as the total number of performed repetitions, compared to conditions without music [21]. All these studies collectively point to the ergogenic effect of listening to preferred music before or during exercise. Potentially, in the long term, listening to preferred music during resistance training may contribute to its greater effectiveness, encompassing strength, power, and muscular endurance in resistance training. However, it is worth noting that, to the best of the authors’ knowledge, the impact of listening to music on performance in lower body multi-joint resistance exercises has not been examined thus far. Additionally, it seems that no studies have been published to directly compare the influence of preferred and non-preferred music on the power output during resistance exercises involving both the upper and lower body, such as BS and BP. Considering that BS is known to elicit higher hemodynamic and metabolic demands than BP [22], the ergogenic effect of music may differ between these exercises.

The effectiveness of music also depends on when it is listening in relation to the task and exercise intensity [23]. While research has explored its impact in resistance training and warm-ups [24,25], little extrapolation can be made to competitive sports where music is prohibited during competitions (e.g., weightlifting). Moreover, there is a gap in evidence regarding the effects of listening to music solely during rest intervals. To the authors’ knowledge, no study has examined the acute effects of music during rest intervals on resistance exercise performance.

Considering shortcomings in the knowledge regarding the acute impact of listening to music on power output during resistance training, the aim of this study was to evaluate the influence of listening to preferred music during active and passive rest intervals on the relative peak power output during BS and BP. It was hypothesized that listening to music during both active and passive rest intervals would enhance the power during BS and BP, with a greater degree of improvement after the former. 

## 2. Materials and Methods

### 2.1. Participants

Thirteen moderately resistance trained [26] healthy males (age: 22 ± 2 years, body mass: 79.9 ± 10.2 kg, body height: 180 ± 6 cm, training experience: 3 ± 2 years, 1RM value in BS: 140 ± 35.4 kg, and 1RM value in BP: 86.5 ± 26.4 kg) and two moderately trained healthy females (age: 23 ± 2 years, body mass: 72 ± 7 kg, body height: 175 ± 1 cm, training experience: 3 ± 2 years, 1RM value in BS: 106.3 ± 12.4 kg, and 1RM value in BP: 52.5 ± 10.6 kg) participated in the study. The inclusion criteria for the study group were as follows: regular participation in resistance training at least 3 times a week for 3 years; no diseases (self-declaration) of the cardiorespiratory system, such as hypertension, atrial fibrillation, thrombosis, or heart failure, and no musculoskeletal injuries for at least 6 months before the examination. Participants were asked not to engage in any resistance exercise 48 h before the start of the experimental session. They were instructed to maintain their regular dietary habits and avoid using any supplements or stimulants before and during the experiment. They were also informed about the potential risks and benefits of participating in the study and were informed that they were free to withdraw from the experiment at any time. All participants gave written consent to participate in the study but were not informed of the expected results. Additionally, each subject performed the experimental sessions individually to avoid competition with other participants. Randomization was performed using the randomization.com generator, assigning each participant a number and determining the order of individual sessions. After being randomly assigned to the training intervention, participants were unaware of the subsequent course of the experiment. The experimental project was approved by the Bioethics Committee for Scientific Research (3/2021; date of approval: 17 June 2021) at the Academy of Physical Education in Katowice, Poland, in accordance with the ethical standards of the Declaration of Helsinki (1983). No participants withdrew from the study.

### 2.2. Experimental Approach to the Problem

The experiment was conducted according to a randomized crossover design where participants participated in one familiarization session and four experimental sessions. This setup was designed to test the effect of listening to preferred music during active and passive rest intervals on relative peak power output during BS and BP. Participants took part in four experimental sessions, which differed in the type of rest interval and lasted 3 min between the sets and 5 min between exercises: (i) passive without music (PNM), (ii) passive with music (PM), (iii) active without music (ANM), (iv) active with music (AM). During each experimental session, participants performed 3 sets of 3 repetitions of BS and BP on a Smith machine (to ensure that the movement trajectory always remains the same) with an external load of 50%1RM [27].

### 2.3. Procedures

#### 2.3.1. Familiarization Session and the One-Repetition Maximum Test 

A week before the start of the experimental sessions, participants conducted a familiarization session. Both the familiarization and the experimental sessions were always held until the first half of the day. During the familiarization session, participants performed an individual warm-up which they also used during each experimental session, and then proceeded to perform a one-repetition maximum (1RM) test where the BS was always performed first, followed by (after a 5 min rest interval) the BP. During each repetition, two individuals experienced in resistance training provided safety and ensured maximum barbell velocity by spotting the participants. Participants were familiar with the 1RM test, as they had previously undergone it as part of their training process to determine the intensity for prescribing the resistance training program.

#### 2.3.2. One-Repetition Maximum Test

After performing an individual warm-up, participants completed 6 warm-up repetitions on an empty barbell and then determined the first test load at the Smith machine, which was increased in each subsequent attempt by 2.5 to 20 kg, depending on the exercise. This process was repeated until an unsuccessful attempt was made. For the BS, the participants began in an upright position, feet shoulder-width apart, with knees and hips fully extended. Feet were flat on the floor, positioned parallel or slightly externally rotated. The barbell rested on the back at the acromion level, ensuring constant contact with the back and shoulders throughout. From this position, they descended until touching the bench, which was defined as when the trochanter major aligned with the upper part of the patella. Concerning the BP, the participants unracked the bar by their own and began the lift with their arms extended and elbows locked. The ‘touch-and-go’ procedure was adopted, in that the bar was required to touch the chest before being pressed to full arm extension [28]. The rest interval between sets was 5 min. The grip width on the barbell was individually determined and used in all attempts during the experimental session. Olympic equipment from (Eleiko International, Halmstad, Sweden) was used in the study (barbell: 2.8 cm diameter; 1.92 m length). During the squat exercise, technical criteria were applied in accordance with Martínez-Cava et al. [29] and according to the rules of the International Powerlifting Federation [30]. During the familiarization session, including the 1RM test, the participants did not listen to music.

#### 2.3.3. Experimental Sessions 

According to randomization, the study participants performed four experimental sessions of squats and bench presses on the Smith machine in random order with a minimum 72 h interval. 

After the same warm-up as during the familiarization session, each participant performed 3 sets of 3 explosive repetitions of BS and BP on the Smith machine with specified rest intervals, depending on the condition: (i) PWM, passive rest interval without music; (ii) PM, passive rest interval with music; (iii) AWM, active rest interval without music; (iv) AM, active rest interval with music.

During the PWM and PM, the participants spent their time in a seated position, while during the AWM and AM, they walked for the entire duration of 3 min. During the PM and AM, each participant listened to their preferred genre of music at their preferred volume and frequency on their own headphones [21]. Heart rate during all sessions was measured using a heart rate monitor (Polar H10, Kempele, Finland).

Participants were instructed to perform each repetition at maximum movement velocity without stopping the barbell at the bottom or top position. A linear position transducer system (Tendo Power Analyzer, Tendo Sport Machines, Trencin, Slovakia) was used to evaluate bar velocity which was then immediately calculated to power output values by the manufacturer software. Previous studies have demonstrated the high reliability and validity of this linear position transducer (intraclass correlation coefficient [ICC] 0.970 to 0.988) [31]. The cable of the sensor module was attached to the end of the barbell using a Velcro strap. The device was positioned in a way that the trajectory of the cable during the movement was as perpendicular to the ground as possible. The accuracy of the measurement was ensured by the same experienced individual who was familiar with the research procedures using the Tendo TM Power Analyzer.

### 2.4. Statistical Analyses

Post hoc power analysis using G*Power version 3.1.9.2 (Dusseldorf, Germany) for the parameters, such as “ANOVA, repeated measures, within factors,” was assumed as a statistical test (1 group of subjects, 4 experimental conditions, and 3 measurements) and the significance level of 0.05 indicated that an effect size of at least 0.32 was needed to achieve a power above 80%. All data were analyzed using IBM SPSS Statistics for Macintosh, Version 25.0 (IBM Corp., Armonk, NY, USA) and were shown as means, with standard deviations (±SD) with their 95% confidence intervals (CI). Statistical significance was set at *p* < 0.05. The normality of data distribution was verified using Shapiro–Wilk tests and Mauchly’s test was used to test the assumption of sphericity. The two-way ANOVAs (4 conditions [PNM; PM; ANM; AM] × 3 sets [set_1; set_2; set_3]) were used to investigate the influence of rest type on relative peak power output during back squat and bench press. Moreover, one-way ANOVAs were used to verify differences in heart rate between conditions. When a significant interaction or main effect was found, the post hoc tests with Bonferroni correction were used to analyze the pairwise comparisons. The magnitude of mean differences was expressed with standardized effect size (ES). Thresholds for qualitative descriptors of Cohen’s d were defined as ≤0.20 a small effect, 0.21 to 0.50 a moderate effect, 0.51 to 0.80 as a large effect, and >0.80 as a very large effect.

## 3. Results

The Shapiro–Wilk test did show a statistically significant violation of data distribution for vastus lateralis frequency.

### 3.1. Bench Press

Two-way ANOVA indicated no statistically significant interaction (F = 2.167; *p* = 0.054; η^2^ = 0.134 and F = 1.756; *p* = 0.118; η^2^ = 0.111) nor the main effect of condition (F = 1.068; *p* = 0.373; η^2^ = 0.071 and F = 0.377; *p* = 0.77; η^2^ = 0.026), but a main effect of the set was found (F = 16.579; *p* < 0.001; η^2^ = 0.542 and F = 22.985; *p* < 0.001; η^2^ = 0.621) for relative mean and peak power output, respectively. The post hoc comparisons showed significantly higher relative mean power output in set_2 (*p* < 0.001; ES = 0.12) and set_3 (*p* = 0.001; ES = 0.16) in comparison to set_1. Similarly, a significantly higher relative peak power output was found in set_2 (*p* < 0.001; ES = 0.10) and set_3 (*p* < 0.001; ES = 0.11) in comparison to set_1 (Table 1).

### 3.2. Back Squat

Two-way ANOVA indicated no statistically significant interaction (F = 0.69; *p* = 0.658; η^2^ = 0.044) nor the main effect of condition (F = 0.579; *p* = 0.632; η^2^ = 0.037), and effect of the set (F = 0.938; *p* = 0.403; η^2^ = 0.059) for relative mean power output. In the case of relative peak power output, there was a non-statistically significant interaction (F = 0.923; *p* = 0.483; η^2^ = 0.062) and a main effect of condition (F = 2.365; *p* = 0.085; η^2^ = 0.145) but a main effect of the set to decrease relative peak power output was found (F = 4.266; *p* = 0.024; η^2^ = 0.234). The post hoc comparisons did not show any significant differences between sets (Table 2).

### 3.3. Heart Rate

**Figure 1:**
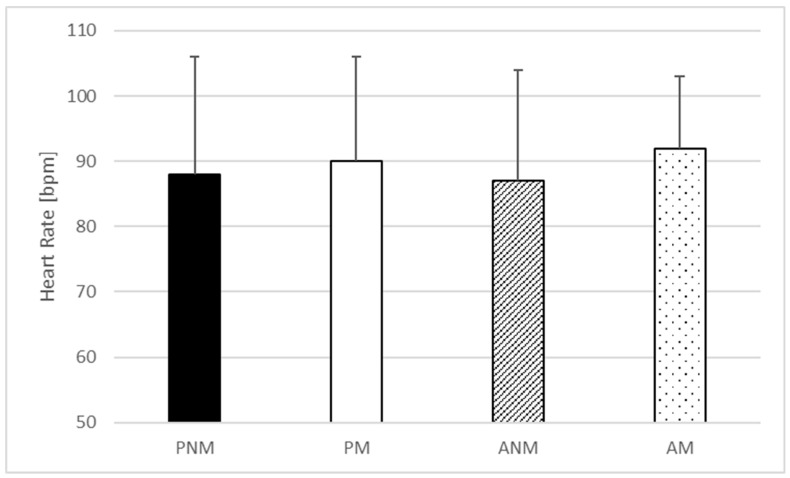
Resting heart rate values. PNM—passive rest without music listening; PM—passive rest with music listening; ANM—active rest without music listening; AM—active rest with music listening.

**Figure 2:**
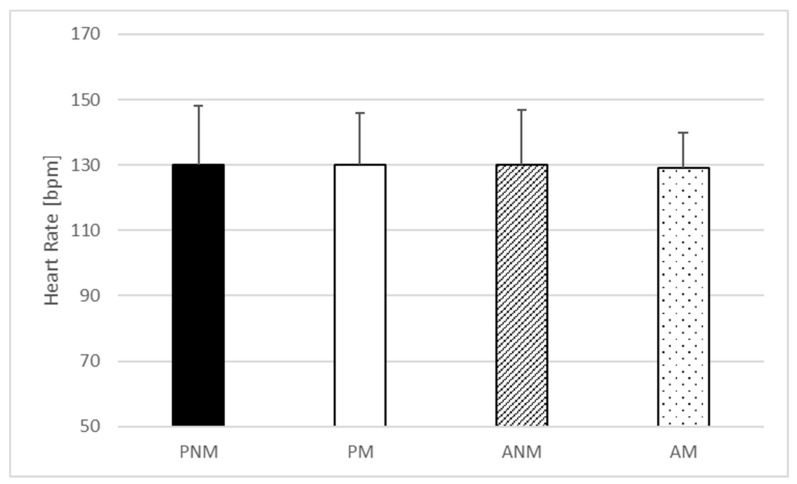
Peak heart rate values. PNM—passive rest without music listening; PM—passive rest with music listening; ANM—active rest without music listening; AM—active rest with music listening.

**Figure 3:**
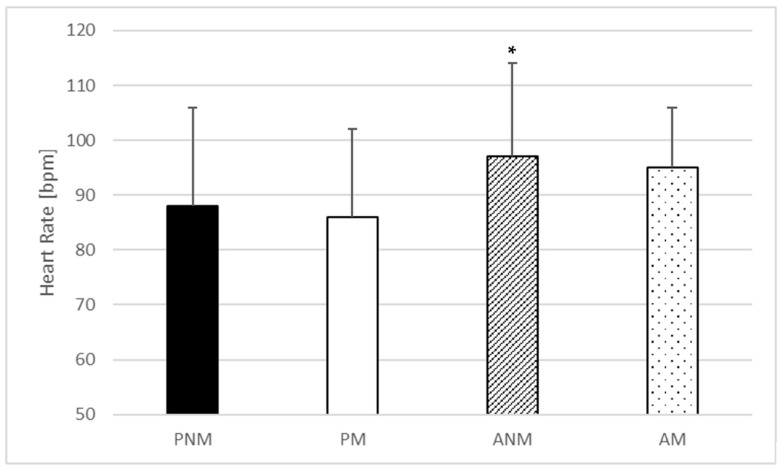
Mean heart rate values. * a significant difference in comparison to passive rest with music listening. PNM—passive rest without music listening; PM—passive rest with music listening; ANM—active rest without music listening; AM—active rest with music listening.

One-way ANOVA indicated no statistically significant effect of the condition in baseline (F = 0.368; *p* = 0.776; η^2^ = 0.026) (Figure 1) and peak heart rate (F = 0.082; *p* = 0.97; η^2^ = 0.006) (Figure 2) but statistically significant differences in the mean heart rate were found (F = 5.246; *p* = 0.004; η^2^ = 0.273) (Figure 3). The post hoc comparisons showed a significantly higher mean heart rate during ANM than in PNM (*p* = 0.043; ES = 0.66) and ANM in comparison to PM condition (*p* = 0.01; ES = 0.78) (Figure 3).

## 4. Discussion

The aim of this study was to assess the impact of listening to preferred music during active and passive rest intervals on relative peak power output during Smith machine BS and BP. The main finding of this study was that music had no effect on the power output in BS and BP exercises. However, a statistically significant decrease in peak power output during subsequent BS sets was revealed for all conditions. On the other hand, an opposite situation was reported in the case of BP, where a statistically significant effect of increasing mean and relative peak power output in particular sets of BP was found. Additionally, an increase in mean heart rate was noted in the ANM compared to PNM and PM conditions.

The results of this study are inconsistent with the findings of previous research indicating the ergogenic effects of listening to music before or during resistance exercises. It seems that the reasons for these differences should be sought in the disparities between the protocols of this study and previous research [19,24]. Firstly, previous studies assumed the performance of only a single upper-body resistance exercise such as bench press or latissimus pulldowns [15,16]. Additionally, they involved only single or two sets with high loads (75–100%1RM) and assessed repetitions performed to volitional failure [21]. Moreover, in the Ballmann et al. study [21], participants also listened to music only before the power output and muscular endurance assessments. In contrast, in this study, participants performed three sets of both BS and BP in a single training session with a load of 50%1RM; however, music was also listened to only during rests. Furthermore, in this study, the exercise order was not randomized, and all participants began the measurement sessions with BS. This may indicate a limited ergogenic effect of listening to music, possibly applying only to upper-body exercises. On the other hand, this study also did not demonstrate the impact of listening to music on power output during BP. Thus, this suggests that the ergogenic effect of music in resistance training might depend on the load and be more pronounced during high-intensity efforts [23]. The mechanisms underlying the observed ergogenic effect of listening to preferred music are not well understood, however, it is suggested that the main one is a modulation of attentional focus [25]. It is possible that, because participants in this study were verbally encouraged to perform each repetition with full engagement, listening to music was not able to provide any additional effect. Moreover, it cannot be ruled out that perhaps the applied load was moderate and the duration of effort was short, thereby not arousing the participants’ need for attentional focus. This is in line with the study by Hutchinson and Tenenbaum [32], which indicates that under conditions of high workload and prolonged duration, attentional focus on overwhelming physiological sensations dominates. At this point, attentional focus becomes almost inevitable. An alternative and likely explanation is that participants received verbal encouragement during exercise, unintentionally fostering a high level of attentional focus. As a result, listening to music may not have further enhanced this focus, leading to a potential “ceiling effect”. In previous studies where the ergogenic effect of music on performance in resistance exercises was reported, participants were not verbally encouraged before performing the exercise [20,21]. Therefore, future research should focus on directly comparing verbal motivation and music, as well as their combined influence on performance in resistance training.

Despite the lack of a meaningful impact of music listening on power performance, a statistically significant power increase in particular sets during bench press for all conditions was reported. Nonetheless, completely different results were obtained in the case of BS, where a significant decrease in power output was noted in successive sets. In the case of BP, these results could potentially be attributed to the post-activation performance enhancement effect, as previously reported in studies analyzing changes in power output between bench press sets [33]. Entirely different results were obtained during back squats, where a significant decrease in power output was observed in particular sets. Considering that BS are known to elicit higher hemodynamic and metabolic demands than BP [22], and the fact that participants in this study were intermediately resistance trained, this could have contributed to a gradual decline in power output due to their limited ability to resist local fatigue [34].

In this study, we noted a statistically significant increase in mean heart rate in the ANM condition compared to the PM and PNM conditions, with a large effect size (ES = 0.78 and 0.66, respectively). Interestingly, no significant differences were found between AM and passive conditions; however, the effect sizes were also moderate to large (ES = 0.49 compared to PNM and ES = 0.61 compared to PM). Therefore, the results from our study showed that listening to music during rest intervals of resistance training has no impact on heart rate. On the other hand, it is also revealing that a very low-intensity physical activity such as walking is able to maintain mean heart rate during long rest intervals (3 min) [35]. Active rest periods stimulate physiological mechanisms, including the cardiovascular response, and thereby enhance readiness for physical exertion [36]. The observed higher mean heart rate in ANM condition might stimulate greater blood flow resulting from low-intensity physical activity during the rest period, which beneficially affects the delivery of oxygen and nutrients to the muscles and the removal of metabolic by-products leading to favorable adaptations [37] which, however, had no effect on the power performance in our study. The higher mean heart rate observed in the ANM in comparison to the PM and PNM conditions partially aligns with the results reported by Thakare et al. [38]. In their study, no correlation was found between the increase in heart rate and the presence or absence of music during treadmill running at self-selected speeds. Patania et al. [39] compared the impact of music on heart rate and the assessment of perceived exertion during endurance exercises, i.e., walking on a treadmill (6 km/h) and high-intensity exercises, i.e., leg press (80%1RM). The results in this study showed greater benefits of listening to music during endurance exercise, where a lower rate of perceived exertion and higher metabolic demand were noted. The authors suggest that anaerobic training, due to its characteristics, requires fewer decision-making processes and is therefore less sensitive to additional external stimuli, which may also explain the results of our research. Spierer et al. [40] demonstrated an increase in total work performed in a repeated Wingate test during the active rest condition, where participants continued to perform low-intensity work on a stationary bike during the rest period compared to a passive rest period. Signorile et al. [41] showed that active recovery provides better performance than passive rest in repeated, short-duration, high-intensity explosive exercises, i.e., eight consecutive 6 s supramaximal rides on a modified cycle ergometer, but this was not reported in our study, which may be related to a different type of effort as well as rest intervals. This study also demonstrated that listening to music during rest intervals in resistance training may potentially have a slight effect in lowering mean heart rate. However, further research is needed to verify this observation, taking into account various music genres.

It is worth noting that there are several methodological limitations that could potentially have influenced the results of this study. Firstly, the volume (beats per minute) and tempo of the music were individually chosen by the participants without standardization. This is noteworthy because previous evidence has shown that volume can affect arousal and change exercise performance [21,42,43]. However, allowing participants to choose the volume and tempo according to their own preferences is more representative of individual habits of using music during resistance training. Following the example of the study by Ballmann et al. [21], it is also worth considering measuring the motivation of participants, which will be directly related to increased satisfaction. Additionally, although the participants were resistance trained, they were not elite athletes. Therefore, it is unclear whether these results can be generalized to experienced elite-level athletes. Furthermore, only one load (50%1RM) was used in this study. In future research, it would be valuable to consider gender differences in a similar sample size, which also constitutes a research limitation. Additionally, longitudinal studies are needed to assess the effects of listening to music and various types of rest intervals on resistance training outcomes, including the need to compare different music genres and exercise intensities.

## 5. Conclusions

The results of this study indicate that listening to music had no effect on the power output during BS and BP as well as on the heart rate. However, the results demonstrated a statistically significant increase of relative and mean peak power in the second and third BP sets in comparison to the first one in all conditions. Completely different results were obtained in the case of BS, where a significant decrease in power output was noted in successive sets. In summary, listening to music during rest intervals in resistance training does not have a significant impact on power output and heart rate during resistance exercises.

## Figures and Tables

**Table 1:** Relative power output descriptive data during bench press exercise.

	Relative Peak Power [W/kg]			Relative Mean Power [W/kg]		
	set_1 (95%CI)	set_2 (95%CI)	set_3 (95%CI)	set_1 (95%CI)	set_2 (95%CI)	set_3 (95%CI)
PNM	6.22 ± 1.55 (5.36–7.08)	6.33 ± 1.64 * (5.42–7.24)	6.36 ± 1.59 * (5.48–7.24)	4.46 ± 1.21 (3.79–5.13)	4.54 ± 1.24 * (3.85–5.23)	4.51 ± 1.20 * (3.85–5.17)
PM	6.04 ± 1.48 (5.22–6.86)	6.28 ± 1.58 * (5.40–7.16)	6.34 ± 1.64 * (5.43–7.25)	4.31 ± 1.10 (3.70–4.92)	4.59 ± 1.26 * (3.89–5.29)	4.48 ± 1.27 * (3.78–5.18)
ANM	6.21 ± 1.67 (5.29–7.13)	6.34 ± 1.68 * (5.41–7.27)	6.35 ± 1.65 * (5.44–7.26)	4.50 ± 1.28 (3.79–5.21)	4.59 ± 1.27 * (3.89–5.29)	4.64 ± 1.27 * (3.94–5.34)
AM	6.20 ± 1.57 (5.33–7.07)	6.37 ± 1.56 * (5.51–7.23)	6.30 ± 1.53 * (5.45–7.15)	4.46 ± 1.20 (3.80–5.12)	4.56 ± 1.19 * (3.90–5.22)	4.60 ± 1.18 * (3.95–5.25)

* a significant difference in comparison to set_1 (*p* < 0.05); PNM—passive rest interval without music; PM—passive rest interval with music; ANM—active rest interval without music; AM—active rest interval with music; CI-confidence interval.

**Table 2:** Relative power output descriptive data during back squat exercise.

	Relative Peak Power [W/kg]			Relative Mean Power [W/kg]		
	set_1 (95%CI)	set_2 (95%CI)	set_3 (95%CI)	set_1 (95%CI)	set_2 (95%CI)	set_3 (95%CI)
PNM	12.51 ± 3.37 (10.64–14.38)	12.21 ± 3.33 (10.37–14.05)	12.10 ± 3.14 (10.36–13.84)	7.49 ± 2.07 (6.34–8.64)	7.44 ± 2.03 (6.32–8.56)	7.34 ± 1.91 (6.28–8.40)
PM	12.16 ± 3.56 (10.19–14.13)	12.20 ± 3.45 (10.29–14.11)	12.09 ± 3.31 (10.26–13.92)	7.43 ± 2.17 (6.23–8.63)	7.48 ± 2.05 (6.34–8.62)	7.45 ± 2.13 (6.27–8.63)
ANM	12.59 ± 3.50 (10.65–14.53)	12.45 ± 3.50 (10.51–14.39)	12.28 ± 3.31 (10.45–14.11)	7.54 ± 2.04 (6.41–8.67)	7.50 ± 2.09 (6.34–8.66)	7.49 ± 1.98 (6.39–8.59)
AM	12.47 ± 3.49 (10.54–14.40)	12.44 ± 3.37 (10.57–14.31)	12.44 ± 3.32 (10.60–14.28)	7.45 ± 2.08 (6.30–8.60)	7.48 ± 2.09 (6.32–8.64)	7.48 ± 2.04 (6.35–8.61)

PNM—passive rest interval without music; PM—passive rest interval with music; ANM—active rest interval without music; AM—active rest interval with music; CI-confidence interval.

## Data Availability

The datasets used and/or analyzed during the current study are available from the corresponding author upon reasonable request.
